# Genome-Wide Association Study Reveals a Novel Association Between MYBPC3 Gene Polymorphism, Endurance Athlete Status, Aerobic Capacity and Steroid Metabolism

**DOI:** 10.3389/fgene.2020.00595

**Published:** 2020-06-16

**Authors:** Fatima Al-Khelaifi, Noha A. Yousri, Ilhame Diboun, Ekaterina A. Semenova, Elena S. Kostryukova, Nikolay A. Kulemin, Oleg V. Borisov, Liliya B. Andryushchenko, Andrey K. Larin, Edward V. Generozov, Eri Miyamoto-Mikami, Haruka Murakami, Hirofumi Zempo, Motohiko Miyachi, Mizuki Takaragawa, Hiroshi Kumagai, Hisashi Naito, Noriyuki Fuku, David Abraham, Aroon Hingorani, Francesco Donati, Francesco Botrè, Costas Georgakopoulos, Karsten Suhre, Ildus I. Ahmetov, Omar Albagha, Mohamed A. Elrayess

**Affiliations:** ^1^Anti-Doping Laboratory Qatar, Doha, Qatar; ^2^UCL-Medical School, London, United Kingdom; ^3^Department of Genetic Medicine, Weill Cornell Medicine-Qatar, Qatar-Foundation, Doha, Qatar; ^4^Department of Computer and Systems Engineering, Alexandria University, Alexandria, Egypt; ^5^College of Health and Life Sciences, Hamad Bin Khalifa University, Doha, Qatar; ^6^Department of Molecular Biology and Genetics, Federal Research and Clinical Center of Physical-Chemical Medicine of Federal Medical Biological Agency, Moscow, Russia; ^7^Department of Biochemistry, Kazan Federal University, Kazan, Russia; ^8^Institute for Genomic Statistics and Bioinformatics, University Hospital Bonn, Bonn, Germany; ^9^Department of Physical Education, Plekhanov Russian University of Economics, Moscow, Russia; ^10^Graduate School of Health and Sports Science, Juntendo University, Chiba, Japan; ^11^Department of Physical Activity Research, National Institutes of Biomedical Innovation, Health and Nutrition, Tokyo, Japan; ^12^Faculty of Health and Nutrition, Tokyo Seiei College, Tokyo, Japan; ^13^Japanese Society for the Promotion of Science, Tokyo, Japan; ^14^Laboratorio Antidoping, Federazione Medico Sportiva Italiana, Rome, Italy; ^15^Department of Physiology and Biophysics, Weill Cornell Medicine-Qatar, Qatar-Foundation, Doha, Qatar; ^16^Research Institute for Sport and Exercise Sciences, Liverpool John Moores University, Liverpool, United Kingdom; ^17^Laboratory of Molecular Genetics, Kazan State Medical University, Kazan, Russia; ^18^Center for Genomic and Experimental Medicine, Institute of Genetics and Molecular Medicine, The University of Edinburgh, Edinburgh, United Kingdom; ^19^Biomedical Research Institute (BRC), Qatar University, Doha, Qatar

**Keywords:** GWAS, SNP, metabolomics, metabolites, elite athletes, endurance

## Abstract

**Background:**

The genetic predisposition to elite athletic performance has been a controversial subject due to the underpowered studies and the small effect size of identified genetic variants. The aims of this study were to investigate the association of common single-nucleotide polymorphisms (SNPs) with endurance athlete status in a large cohort of elite European athletes using GWAS approach, followed by replication studies in Russian and Japanese elite athletes and functional validation using metabolomics analysis.

**Results:**

The association of 476,728 SNPs of Illumina DrugCore Gene chip and endurance athlete status was investigated in 796 European international-level athletes (645 males, 151 females) by comparing allelic frequencies between athletes specialized in sports with high (*n* = 662) and low/moderate (*n* = 134) aerobic component. Replication of results was performed by comparing the frequencies of the most significant SNPs between 242 and 168 elite Russian high and low/moderate aerobic athletes, respectively, and between 60 elite Japanese endurance athletes and 406 controls. A meta-analysis has identified rs1052373 (GG homozygotes) in Myosin Binding Protein (*MYBPC3*; implicated in cardiac hypertrophic myopathy) gene to be associated with endurance athlete status (*P* = 1.43 × 10^−8^, odd ratio 2.2). Homozygotes carriers of rs1052373 G allele in Russian athletes had significantly greater VO_2__*max*_ than carriers of the AA + AG (*P* = 0.005). Subsequent metabolomics analysis revealed several amino acids and lipids associated with rs1052373 G allele (1.82 × 10^–05^) including the testosterone precursor androstenediol (3beta,17beta) disulfate.

**Conclusions:**

This is the first report of genome-wide significant SNP and related metabolites associated with elite athlete status. Further investigations of the functional relevance of the identified SNPs and metabolites in relation to enhanced athletic performance are warranted.

## Background

Elite athletic performance is a multi-factorial trait with input from both genetic and environmental factors. The superior performance of elite athletes has been historically considered an outcome of a special talent shaped by intensive training. The talent is now believed to be a product of additive genetic components predisposing the athlete to endurance, speed, strength, flexibility and coordination trainability under the control of strong environmental cues including exercise and nutrition. In this model, the genetic predisposition together with ability to respond to training are the keys to the superior physical performance of elite athletes (Georgiades et al., 2017).

Sports can be classified according to the type and intensity of the exercise required to perform during competition. The percentage of maximal oxygen uptake (VO_2__*max*_) is a detrimental factor in the categorization of endurance sports, as it reflects the maximal cardiac output, the oxygen transport capacity, and the blood volume (Bergh et al., 2000). Accordingly, sports can be divided into sport events with low, moderate and high aerobic (dynamic) component (Mitchell et al., 2005). Similarly, the percent of maximal voluntary contraction (MVC), which reflects the greatest amount of tension a muscle can generate and hold, is used to classify sports into sporting disciplines with low, moderate and high power component (Mitchell et al., 2005).

Classical twin and family genetic studies have suggested that VO_2__*max*_ is up to 94% inherited (Bouchard et al., 1998; Peeters et al., 2009). Genome-wide association studies (GWAS) in athletes versus non-athletes have uncovered many new loci in association with VO_2__*max*_ (Rankinen et al., 2010; Bouchard et al., 2011) and elite endurance performance (Ahmetov et al., 2015). A more recent review of genetic predisposition to elite athletic endurance has highlighted 100 endurance variants (Semenova et al., 2019). However, despite some initial evidence suggesting identification of genetic variants in GWAS studies, further studies did not replicate/validate these findings hindered by a small sample size and complex phenotype (Pitsiladis et al., 2016). One of the first GWAS in athletes using 143 K single-nucleotide polymorphisms (SNPs) and subsequent meta-analysis of 45 promising genetic markers in 1,520 endurance athletes and 2,760 controls has revealed only one statistically significant marker (rs558129 at *GALNTL6*) associated with endurance status in world class athletes, but not at genome wide level of significance (Rankinen et al., 2016). Therefore, the genetic predisposition to endurance traits remains unclear, largely due to the relatively underpowered elite athletes’ cohorts. Recently, a polymorphism in human homeostatic iron regulator protein was found to be associated with elite endurance athlete status and aerobic capacity in Russian athletes (Semenova et al., 2020).

Metabolomics analysis has presented a novel tool to validate genomics data by providing an intermediate phenotype (metabolites) in association with the identified genetic variants (Kastenmuller et al., 2015; Tanaka et al., 2016). Pilot metabolomics studies have revealed differences in the metabolic signature of moderate and high endurance elite athletes, such as steroid biosynthesis, fatty acid metabolism, oxidative stress and energy-related molecular pathways (Al-Khelaifi et al., 2018, 2019a). Recently, a study investigating metabolic GWAS of elite athletes showed novel genetically influenced metabolites associated with athletic performance. These included two novel genetic loci in FOLH1 and VNN1 in association with N-acetyl-aspartyl-glutamate and linoleoyl ethanolamide, respectively, and one novel locus linking genetic variant in SULT2A1 and androstenediol (3alpha, 17alpha) monosulfate in endurance athletes (Al-Khelaifi et al., 2019b).

In this study, we aimed to investigate the association of multiple SNPs and endurance athlete status in a relatively large cohort of European elite athletes specialized in sports with high and low/moderate aerobic component using GWAS approach and replicate our findings in elite Russian and Japanese athletes. We also aimed to perform functional validation using VO_2__*max*_ testing and metabolomics analysis by identifying metabolites that are associated with significant endurance-related SNPs.

## Results

### Genome-Wide Association Study

Athletes from the discovery cohort were classified into different groups of sports following previously published sports classification criteria (Mitchell et al., 2005), as shown in Table 1.

**TABLE 1:** Classification of GWAS participants according to sports classes.

	Low/moderate (<70% VO_2_max)		High (>70% VO_2_max)			Total
High (>50% MVC)	Wrestling and Judo (8M)	Skate boarding (2M)	Modern Pentathlon (1F)			287
			Kayaking (1F)	Rowing (9M/8F)	Biathlon (2M/1F)	
	Weightlifting (14M/7F)		Boxing (4M/7F)	Cycling (157M/49F)	Triathlon (8M/9F)	
Moderate	Jumping (athletics) (1F)		Handball (19M/3F)	Skiing Cross Country (3M/1F)	Basketball (3M)	165
(20–50% MVC)	Rugby (15M)	Aquatics (3M/2F)				
	Athletics other (41M/26F)	Sprint (2M)	Hockey (4M/1F)		Swimming (25M/16F)	
Low (<20% MVC)	Baseball (2M)		Long-Distance running and marathon (37M/12F)		Tennis (3M/3F)	344
	Volleyball (2M)					
	Table tennis (9M)		Soccer (256M/1F)	Ultra-running (1F)	Football (17M/1F)	
	134		662			796

*Distribution of elite athletes in various categories based on sport type-associated peak dynamic (maximal oxygen uptake percentage; VO_2*max*_) and peak static (maximal voluntary muscle contraction percentage; MVC) components achieved during competition as described previously (Mitchell et al., 2005).*

**FIGURE 1:**
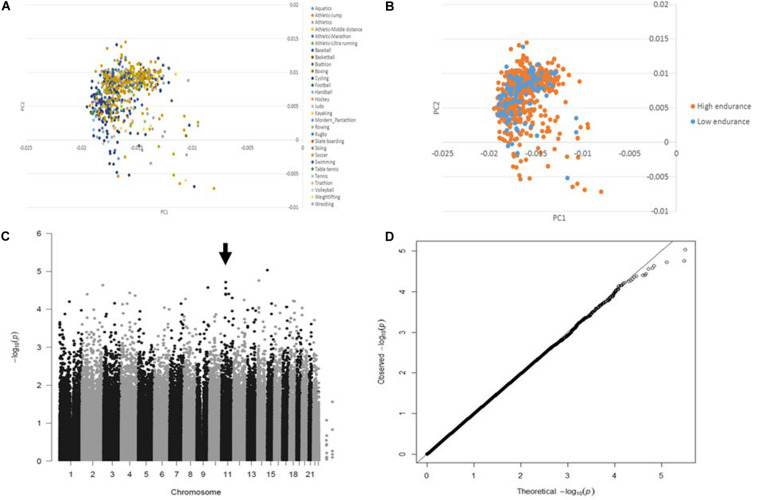
GWAS data quality control. PCA shows no difference in the genotype distribution among sport disciplines **(A)** or between groups (sports with low/moderate versus high aerobic component) **(B)** Manhattan (arrow indicates significant SNPs) **(C)** and Quantile-quantile (no evidence of genomic inflation, lambda GC = 1.006) **(D)** plots illustrating GWAS results in association with endurance.

The principle component analysis (PCA) of the genotyping data revealed no influence of sport disciplines (Figure 1A) or training modality (i.e., sports with low/moderate versus high aerobic component) (Figure 1B) on genotype distribution. Following quality control data processing, genotyping of 341385 SNPs in 796 European elite athletes revealed several variants associated with endurance athlete status, but none reached GWAS level of significance. Table 2 shows top SNPs (*P* < 5 × 10^−5^) with their odd ratios (OR) in relation to elite athletic endurance, location according to function genome variation server (GVS), gene name and minor allele frequency (MAF) in sports with high and low/moderate aerobic component. MAF in non-elite athletes from 1,000 genome project were used as a reference. Figure 1 shows Manhattan (C) and quartile-quartile (QQ) plots (D) of GWAS hits associated with endurance.

**TABLE 2:** Top GWAS SNPs associated with endurance athlete status from the discovery study.

rsID	Chromosome	Position	Reference base	*N*	OR	Standard error	*P* value	Function GVS	Gene list	MAF-high aerobic *N* = 662	MAF- moderate/low aerobic *N* = 134	MAF-non-athletes
rs8029108	15	22945314	C	795	0.5293	0.1435	9.23 × 10^–6^	intron	CYFIP1	0.4448	0.403	*G* = 0.36
kgp5680198	14	34627202	C	792	0.5151	0.1545	1.75 × 10^–5^	intergenic	LOC102724945	0.2135	0.3246	*C* = 0.27
rs10838681	11	47275064	A	794	0.5208	0.1526	1.92 × 10^–5^	intron	NR1H3	0.233	0.3496	*A* = 0.35
kgp2861067	2	234653039	T	795	0.2227	0.3551	2.34 × 10^–5^	intron	UGT1A10	0.01815	0.0597	*T* = 0.013
kgp11512684	9	123798492	A	793	0.202	0.3808	2.66 × 10^–5^	intron	C5	0.01364	0.04887	*A* = 0.016
rs1052373	11	47354787	A	796	0.5393	0.1475	2.81 × 10^–5^	missense	MYBPC3	0.2764	0.3955	*T* = 0.39
rs17020631	4	94380515	G	795	0.3064	0.2866	3.68 × 10^–5^	intron	GRID2	0.0287	0.09398	*G* = 0.09
rs1949886	11	80311066	A	796	4.346	0.3573	3.92 × 10^–5^	intergenic	none	0.1329	0.03731	*A* = 0.15
rs7120118	11	47286290	C	796	0.5455	0.1475	3.97 × 10^–5^	intron	NR1H3	0.2696	0.3881	*C* = 0.38

### Replication of Endurance SNPs in Russian and Japanese Elite Athlete Cohorts

Replication of results was performed by comparing the frequencies of the most significant SNPs (*P* < 10^−5^) in 242 elite Russian high and 168 low/moderate aerobic athletes, and in 60 elite Japanese endurance athletes and 406 controls. Out of the 9 top SNPs identified form the GWAS discovery stage, the rs1052373 (*MYBPC3*) and rs7120118 (*NR1H3*) showed significant association with endurance in Russian and Japanese (*P* < 0.05). However, the association was driven by a dominant model since results of this analysis showed over representation for rs1052373 GG and rs7120118 TT genotypes in the high endurance group. A subsequent meta-analysis has confirmed the over representation of the rs1052373 GG and rs7120118 TT genotypes in high endurance sports at genome-wide and Bonferroni levels of significance (1.43 × 10^–8^ and 1.66 × 10^–7^, respectively) (Table 3). The combined analysis showed no evidence of heterogeneity and direction of association was similar in all three cohorts.

**TABLE 3:** SNPs associated with Endurance athlete status from the discovery, replication and meta-analysis.

Chr	SNP	RG	GWAS		Russian		Japanese		Combined			
			*P*	OR (95% CI)	*P*	OR (95% CI)	*P*	OR (95% CI)	*P*	OR (95% CI)	*I*^2^	*P*_*het*_
11	rs1052373	GG	5.48 × 10^–6^	2.61 (1.72–3.94)	0.012	1.67 (1.12–2.49)	0.0027	2.92 (1.41–6.05)	1.43 × 10^–8^	2.17 (1.67–2.84)	35	0.2
11	rs7120118	TT	1.26 × 10^–5^	2.49 (1.65–3.75)	0.016	1.64 (1.10–2.45)	0.0352	2.48 (1.10–5.56)	1.66 × 10^–7^	2.07 (1.59–2.70)	12	0.3

*OR, odds ratio for the risk genotype; CI, confidence interval; *I*^2^, heterogeneity statistics; *P*_*het*_, *P* value for heterogeneity.*

**FIGURE 2:**
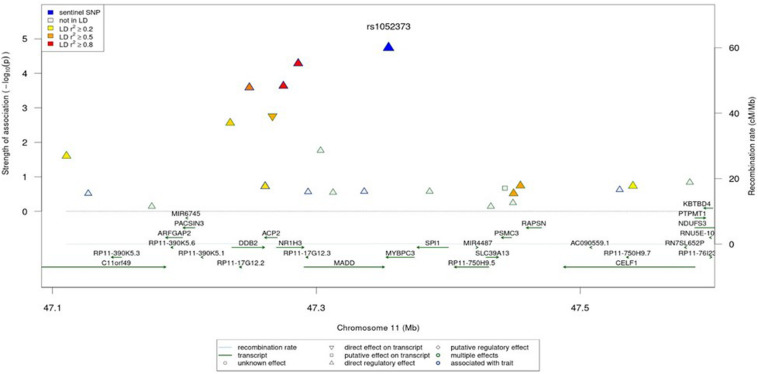
Regional association plot for the region around rs1052373. The colors correspond to different LD thresholds, where LD is computed between the sentinel SNP (lowest *P*-value, colored in blue) and all SNPs. Shapes of markers correspond to their functionality as described in the legend.

The regional association plot for the rs1052373 G allele in *MYBPC3* gene revealed a number of SNPs in the same LD block in association with high endurance including the rs7120118 T allele in NR1H3 gene (Figure 2).

To validate the potential functionality of the identified GWAS SNPs, association of the identified two SNPs (rs1052373 G and rs7120118 T alleles) with VO_2__*max*_ was investigated in a subgroup of the Russian replication cohort in which VO_2*max*_ data was available. This included 32 elite Russian long-distance athletes [19 biathletes, 13 cross-country skiers; 17 females, age 23.5 (3.5) years; 15 males, age 21.3 (4.1) years]. The rs1052373 GG carriers had significantly greater VO_2__*max*_ than carriers of the AA + AG (*P* = 0.005 adjusted for sex). Similarly, rs7120118 TT carriers showed a trend of higher VO_2__*max*_ than carriers of the CC + CT (*P* = 0.053 adjusted for sex).

**FIGURE 3:**
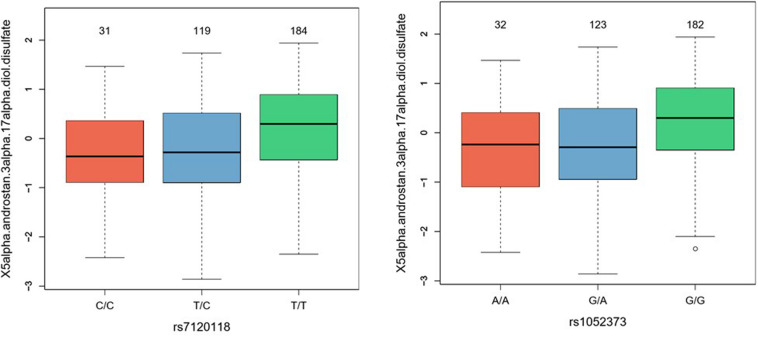
Boxplots representing levels of 5alpha-androstan-3alpha,17alpha-diol disulfate in rs7120118 and rs1052373 genotype groups.

For further validation of the potential functionality of the identified GWAS SNPs, metabolomics of 750 metabolites was carried out in a subset of the discovery cohort (*n* = 490) and enriched metabolic pathways associated with the rs1052373 G allele and rs7120118 T alleles were determined (Table 4). Among the metabolic pathways associated with rs56330321 and rs7120118, various lipids and amino acids were significantly altered by their genotypes. However, only 5alpha-androstan-3alpha,17alpha-diol disulfate reached Bonferroni level of significance (Table 4), exhibiting higher levels in rs1052373 GG and rs7120118 TT carriers compared to AA + AG and CC + TC carriers, respectively (Figure 3).

**TABLE 4:** Metabolites that belong to the significantly enriched phospholipids pathway Top metabolites associated with significant SNPs.

SNP	Beta	SE.Beta	*P*	Metabolites	SUPER_PATHWAY	SUB_PATHWAY
rs1052373	–0.36	0.08	1.82 × 10^–5^	5alpha-androstan-3alpha,17alpha-diol disulfate	Lipid	Androgenic steroids
	–0.25	0.07	0.000248	2-hydroxy-3-methylvalerate	Amino Acid	Leucine, Isoleucine and Valine Metabolism
	–0.23	0.07	0.000879	alpha-hydroxyisovalerate	Amino Acid	Leucine, Isoleucine and Valine Metabolism
	0.31	0.09	0.000928	xylose	Carbohydrate	Pentose Metabolism
	–0.23	0.07	0.001226	N1-methylinosine	Nucleotide	Purine Metabolism, (Hypo)Xanthine/Inosine containing
	–0.23	0.07	0.001315	palmitoleoylcarnitine (C16:1)*	Lipid	Fatty Acid Metabolism(Acyl Carnitine)
	–0.23	0.07	0.001509	2-hydroxyadipate	Lipid	Fatty Acid, Dicarboxylate
	–0.22	0.07	0.001516	2-methylcitrate/homocitrate	Energy	TCA Cycle
	–0.21	0.07	0.001933	myristoleoylcarnitine (C14:1)*	Lipid	Fatty Acid Metabolism(Acyl Carnitine)
rs7120118	–0.33	0.08	5.17 × 10^–5^	5alpha-androstan-3alpha,17alpha-diol disulfate	Lipid	Androgenic Steroids
	–0.27	0.07	0.000136	2-hydroxy-3-methylvalerate	Amino Acid	Leucine, Isoleucine and Valine Metabolism
	–0.24	0.07	0.000582	alpha-hydroxyisovalerate	Amino Acid	Leucine, Isoleucine and Valine Metabolism
	–0.24	0.07	0.000715	N1-methylinosine	Nucleotide	Purine Metabolism, (Hypo)Xanthine/Inosine containing
	0.31	0.09	0.001004	xylose	Carbohydrate	Pentose Metabolism
	–0.23	0.07	0.001527	2-hydroxyadipate	Lipid	Fatty Acid, Dicarboxylate
	0.28	0.09	0.001966	5-acetylamino-6-formylamino-3-methyluracil	Xenobiotics	Xanthine Metabolism
	–0.22	0.07	0.002116	alpha-hydroxyisocaproate	Amino Acid	Leucine, Isoleucine and Valine Metabolism
	–0.22	0.07	0.002216	2-methylcitrate/homocitrate	Energy	TCA Cycle
	–0.22	0.07	0.002266	glycerol	Lipid	Glycerolipid Metabolism

## Discussion

Genetic predisposition into cardiorespiratory fitness and response to exercise training has been previously described (Lortie et al., 1982; Prud’homme et al., 1984; Hamel et al., 1986; Bouchard et al., 1994, 1998, 1999). Since endurance performance sports are characterized by increased cardiorespiratory capacity, genetic predisposition into elite endurance performance is also expected to be genetically influenced (Guth and Roth, 2013). However, genetic studies of elite athletic endurance showed inconsistent results (Guth and Roth, 2013; Ahmetov and Fedotovskaya, 2015; Pitsiladis et al., 2016; Wang et al., 2016). The aims of this study were to carry out the largest GWAS study of elite European athletes to date using a unique SNP microarray that is enriched with genes involved in different metabolic pathways with direct influence on various physiological pathways characteristic of elite athletes. GWAS results have revealed a number of novel SNPs associated with endurance but none reached the GWAS level of significance. Replication of the top identified SNP associations in two independent cohorts of elite athletes from Russia and Japan has confirmed the association of rs7120118 and rs1052373 with endurance athlete status. Subsequent meta-analysis of the three cohorts has revealed for the first time that both SNPs were associated with endurance athlete status at genome-wide and Bonferroni level of significance, respectively. Functional validation has revealed the association of the two SNPs with increased Vo_2__*max*_ and levels of the testosterone precursor 5alpha-androstan-3alpha,17alpha-diol disulfate.

The top identified GWAS significant SNP (rs1052373) is located within *MYBPC3* gene. *MYBPC3* codes for a myosin-associated protein expressed in the cross-bridge-bearing zone (C region) of A bands in striated muscle. The phosphorylation of MYBPC3 protein modulates cardiac contraction (Moss et al., 2015). Mutations in *MYBPC3* were previously associated with a lower super-relaxed state in patients with hypertrophic cardiomyopathy (HCM) (McNamara et al., 2017). Intense exercise can trigger heart remodeling to compensate for the elevations in blood pressure or volume by increasing muscle mass. Hence, hearts of the endurance athletes typically exhibit an eccentric cardiac hypertrophy with increased cavity dimension and wall thickness (Pelliccia et al., 1991; Hedman et al., 2015), which is influenced by the type of sport performed (Pelliccia, 1996; Pelliccia et al., 1999; Maron and Pelliccia, 2006). As a result, the endurance-trained heart can deliver a large maximal systolic volume (35% larger than untrained heart) in order to produce a large cardiac output (Ogawa et al., 1992; Pelliccia et al., 1999). Since carriers of the GG allele exhibit a benign phenotype of HCM according to NIH’s ClinVar database (Landrum et al., 2018), the mild phenotype may be enhancing exercise-triggered physiological adaptations. The seemingly dominant effect of rs1052373 GG on increased VO_2__*max*_ and endurance may support this added advantage although more studies are needed to confirm this finding. These adaptations, however, might be associated with a greater risk of cardiovascular disease. Indeed, we have recently shown that endurance athletes with high cardiovascular demand (higher blood pressure and stroke volume) show metabolic signature consistent with higher risk of cardiovascular disease (Al-Khelaifi et al., 2019a). When investigating the expression quantitative trait loci (eQTLs) associated with rs1052373, a number of genes was identified including SPI1, MYBPC3, MADD, ACP2 and NR1H3 (Ray et al., 1990; Tang and Chu, 2002; Mannan et al., 2004; Wu et al., 2012; Carrier et al., 2015; Theofilopoulos and Arenas, 2015). Interestingly, eQTL (GTEx) showed that rs1052373 polymorphism is associated with expression level of MADD and ACP2 in heart, but not MYBPC3. Since MAP kinase plays an important role of cardiac hypertrophy (Zhang et al., 2003), the association between rs1052373 polymorphism and VO_2__*max*_ and endurance may also be explained by MADD expression, although this needs further validatoin. Information related to function and associated diseases with these genes are summarized in Supplementary Table S1.

The other significant association was between rs7120118 TT carriers and high endurance. Rs7120118 is located in *NR1H3* gene that codes for a nuclear receptor regulating macrophage function, lipid homeostasis and inflammation. NR1H3, also known as liver X Receptor Alpha (LXRA), plays an important role in the regulation of cholesterol homeostasis including adrenal steroidogenesis (Repa et al., 2002; Cummins et al., 2006). The association of rs7120118 with high endurance could be reflecting the high linkage disequilibrium (*r*^2^ = 0.89, *P* < 0.0001) between rs7120118 TT and the potentially functional rs1052373 GG. It could, however, be related to increased synthesis of the testosterone precursor 5alpha-androstan-3alpha,17alpha-diol disulfate since NR1H3 regulates hypothalamo-pituitary–adrenal steroidogenesis (Handa et al., 2011). Indeed, we have previously shown that high-endurance athletes exhibit elevated levels of several sex hormone steroids involved in testosterone synthesis including 5alpha-androstan-3alpha,17alpha-diol disulfate (Al-Khelaifi et al., 2018) with implication on improving performance due to enhanced glucose metabolism and protein synthesis in the muscle (Sato et al., 2008). The functional relevance of these associations remains to be further validated.

Study limitations: The lack of information about participants and the heterogeneity of their sport groups were major limitations of this study. To overcome these limitations and to increase the power of the study, genotyping was compared between athletes who belong to high endurance versus moderate endurance performance sports instead of power versus endurance due to the overlap between the two classes as per Mitchell’s categorization (Mitchell et al., 2005). Other limitations included using add-on replication studies (Russian and Japanese cohorts) rather than using a carefully designed replication. However, differences were confirmed in each study separately and the subsequent meta-analysis confirmed the significance of the association of the two SNPs with endurance.

## Conclusion

This study reports the first GWAS significant SNP (rs1052373) in *MYBPC3* in association with endurance athlete status with a direct relevance to cardiac hypertrophy and contraction. The SNP is associated with increased VO_2*max*_ and elevated levels of the testosterone precursor androstenediol (3beta,17beta) disulfate, both phenotypes that potentially contribute to the superior performance of endurance athletes. This study also identifies a second SNP (rs7120118) associated with endurance at Bonferroni level of significance in *NR1H3.* This SNP could be either working independently of rs1052373 through influencing steroidogenesis or could be acting as a marker of rs1052373. Further investigations of the functional relevance of the identified SNPs and associated metabolites in relation to enhanced athletic performance are warranted.

## Methods

The aim of this study is to investigate the genetic predisposition to elite athletic endurance through conducting the largest GWAS in elite athletes to date, followed by functional validation through aerobic capacity testing and metabolomics analysis to shed light on the underlying mechanisms of genetic associations.

### Participants

#### Discovery Study

Seven hundred and ninety six consented European international-level athletes (645 males, 151 females) from different sports disciplines who participated in national or international sports events and tested negative for doping substances at anti-doping laboratories in Qatar (ADLQ) and Italy (FMSI) were included in this study. No other information of participants was available due to the strict anonymization process undertaken by the anti-doping laboratories. This study was performed in line with the World Medical Association Declaration of Helsinki – Ethical Principles for Medical Research Involving Human Subjects. All protocols were approved by the Institutional Research Board of ADLQ (F2014000009). Athletes were dichotomized into groups with different aerobic (dynamic) and power (static) components (Table 1) based on their sport types as described previously (Mitchell et al., 2005). Table 1 further lists the number of participants based on various analyses as per sport type in each class/group and their genders.

#### Replication Studies

The first replication study involved 410 Russian athletes [187 females, age 25.3 (4.1) years, 223 males, age 25.7 (4.3) years]. Athletes were dichotomized into two groups with different aerobic (dynamic) and power (static) components based on their sport types. Group 1 (242 athletes with high aerobic component) included biathletes (*n* = 19), cross-country skiers (*n* = 16), 800–10,000 m runners (*n* = 9), rowers (*n* = 9), kayakers (*n* = 30), canoers (*n* = 8), speed skaters (*n* = 12), short-trackers (*n* = 3), swimmers (*n* = 38), cyclists (*n* = 5), race walkers (*n* = 6), boxers (*n* = 43), badminton players (*n* = 11), basketball players (*n* = 6), water polo players (*n* = 12), football players (*n* = 9), and ice hockey players (*n* = 6). Group 2 (168 athletes with low aerobic component) included 100–400 m runners (*n* = 8), wrestlers (*n* = 44), alpine skiers (*n* = 2), sailors (*n* = 2), synchronized swimmer (*n* = 1), taekwondo athletes (*n* = 5), baseball players (*n* = 10), volleyball players (*n* = 19), table tennis players (*n* = 5), softball players (*n* = 5), rhythmic gymnasts (*n* = 7), chess players (*n* = 5), throwers (*n* = 6), athletics jumpers (*n* = 16), ski jumpers (*n* = 2), weightlifters (*n* = 25), ure skaters (*n* = 6). All athletes were Olympic team members (International level; all Caucasians of Eastern European descent) who have tested negative for doping substances. The Russian study was approved by the Ethics Committee of the Federal Research and Clinical Center of Physical-chemical Medicine of the Federal Medical and Biological Agency of Russia. Written informed consent was obtained from each participant. The study complied with the guidelines set out in the Declaration of Helsinki and ethical standards in sport and exercise science research. The experimental procedures were conducted in accordance with the set of guiding principles for reporting the results of genetic association studies defined by the STrengthening the REporting of Genetic Association studies (STREGA) Statement.

The second replication study involved endurance athletes (*n* = 60) and controls (*n* = 406) from Japan. All endurance athletes were track and field competitors who participated in endurance events from 800 m to marathon. In addition, all athletes were international athletes who had competed at major international competitions. All controls were healthy Japanese individuals. All subjects gave written informed consent before their inclusion in the study. The study protocols were approved by the ethics committee of the Juntendo University and was conducted according to the Declaration of Helsinki.

### Aerobic Capacity Testing

VO_2*max*_ in biathletes and cross-country skiers was determined using an incremental test to exhaustion on a treadmill HP Cosmos (Germany). The initial speed was 7 km/h, the increment was 0.1 km/h every 10 s. $\dot{\text{V}}$O_2*max*_ was determined breath by breath using a MetaMax 3B-R2 gas analysis system. $\dot{\text{V}}$O_2*max*_ was recorded as the highest mean value observed over a 30 s period.

### Genotyping

#### Discovery Study

DNA was extracted from leukocytes (venous blood) samples from all participants using DNeasy Blood & Tissue kit (Qiagen) following manufacturer’s instructions. The concentration and the quality of DNA were assessed using the Nanodrop (Thermo Fisher) and Qubit Fluorometer (Invitrogen) to ensure sufficient amount and quality of DNA were obtained for genotyping. Illumina Drug Core array-24 BeadChips was chosen for the genotyping of 476,728 SNPs in the 796 European elite athletes collected for Anti-Doping analysis (discovery cohort). This array contains over 240,000 highly-informative genome-wide tag SNPs and a novel ∼200,000 custom marker set designed to support studies of drug target validation and treatment response. The assay required 200 ng of DNA sample as input with a concentration of at least 50 ng/μl. All further procedures were performed according to the instructions of Infinium HD Assay according to manufacturer’s instructions. Briefly, 4 μl of obtained DNA was mixed with Illumina amplification reagents and incubated overnight at 37^*o*^C in hybridization oven. On the second day, enzymatic reagents were used to fragment the amplified DNA then precipitated by centrifugation. Subsequently, re-suspended pellet was loaded in the beadchip then incubated overnight at 48^*o*^C in hybridization oven. On third day, beadchips underwent enzymatic base extension and fluorescent staining. Lastly, after coating, the beadchips were imaged using iScan.

#### Replication Studies

Molecular genetic analysis in Russian cohorts was performed with DNA samples obtained from leukocytes (venous blood). Four ml of venous blood were collected in tubes containing EDTA (Vacuette EDTA tubes, Greiner Bio-One, Austria). Blood samples were transported to the laboratory at 4°C and DNA was extracted on the same day. DNA extraction and purification were performed using a commercial kit according to the manufacturer’s instructions (Technoclon, Russia) and included chemical lysis, selective DNA binding on silica spin columns and ethanol washing. Extracted DNA quality was assessed by agarose gel electrophoresis at this step. HumanOmni1-Quad BeadChips (Illumina Inc, United States) were used for genotyping of 1,140,419 SNPs in athletes and controls. The assay required 200 ng of DNA sample as input with a concentration of at least 50 ng/μl. Exact concentrations of DNA in each sample were measured using a Qubit Fluorometer (Invitrogen, United States). All further procedures were performed according to the instructions of Infinium HD Assay. For the second replication study, total DNA was isolated from saliva or venous blood using Oragene⋅DNA Collection Kits (DNA genotek, Ontario, Canada) or QIAamp DNA blood Maxi Kit (QIAGEN, Hilden, Germany), respectively. The total DNA content was measured using a NanoDrop 8000 spectrophotometer (Thermo Fisher Scientific, MA, United States). Subsequently, DNA samples were adjusted to a concentration of 50 ng/μL with TE buffer and were stored at 4°C. Total DNA samples were genotyped for more than 700,000 markers using the Illumina^®^ HumanOmniExpress Beadchip.

### Data Extraction and SNP Identification

Raw data was extracted, peak-identified and QC processed using Illumina iScan hardware and software. These systems are built on a web-service platform utilizing Microsoft’s NET technologies, which run on high-performance application servers and fiber-channel storage arrays in clusters to provide active failover and load-balancing.

### Metabolomics

Screening of serum metabolites was performed in 490 elite athletes (Supplementary Table S2) using protocols established at Metabolon, Durham, NC, United States. The platform utilizes Waters ACQUITY ultra-performance liquid chromatography (UPLC) and a Thermo Scientific Q-Exactive high resolution/accurate mass spectrometer interfaced with a heated electrospray ionization (HESI-II) source and Orbitrap mass analyzer operated at 35,000 mass resolution. Detailed protocol and QC measures were previously published (Evans et al., 2009; Al-Khelaifi et al., 2018).

### Statistical Analysis

Following genotyping using Illumina’s Drug Core SNP array, analysis was performed using Plink v1.9. Quality control measures were applied to the genotype data set to exclude samples with low genotype call rate or excess heterozygosity. Accordingly, SNPs with a genotype call rate <98%, minor allele frequency <1%, or deviating from Hardy-Weinberg equilibrium (*P* < 10^–6^) were excluded. After filtering the data with the above criteria, 341,385 SNPs were used in analysis. Population background was determined using principal component analysis (PCA) in comparision to samples from HapMap project and only samples with European ancestry were included in the analysis. The analysis in European and Russian cohorts was performed using linear or logistic regression models. A model incorporating sports grouped by training modalities (i.e., sports with high versus low/moderate aerobic component) was used for the discovery cohort after incorporating gender and PCA components 1, 2, 3 & 4 as covariates in the model. A stringent Bonferroni level of significance of *P* ≤ 0.05/341385 = 1.46 × 10^–7^ was used to define significant associations. To perform the meta-analysis, the Cochrane Review Manager version 5.3 was used. Random and fixed effect models were applied. The heterogeneity degree between the studies was assessed with the I^2^ statistics. Associations between SNPs and metabolite levels were computed using lm function in R (version 3.3.1) while correcting for gender, hemolysis and PCA. An additive inheritance model was used (SNPs were coded as 0,1,2 according to their genotype group. Pathway enrichment analyses were carried out using Chi square tests to identify pathways with enriched metabolites ranked by *P*-value from the linear model since Bonferroni level of significance was not observed.

## Data Availability Statement

The SNP data supporting this study is available at: https://figshare.com/articles/GWAS_elite_endurance_athletes/12199760. Summary statistics will be made available through the NHGRI-EBI GWAS Catalog: https://www.ebi.ac.uk/gwas/downloads/summary-statistics.

## Ethics Statement

This study was performed in accordance with the World Medical Association Declaration of Helsinki. All protocols were approved by the Institutional Research Board of anti-doping lab Qatar (F2014000009). The patients/participants provided their written informed consent to participate in this study.

## Author Contributions

All authors contributed to sample collection, analysis, manuscript writing, and manuscript review and acceptance of final version. ME is responsible for the integrity of the work as a whole.

## Conflict of Interest

The authors declare that the research was conducted in the absence of any commercial or financial relationships that could be construed as a potential conflict of interest.

## Abbreviations

ACP2, acid phosphatase 2: Lysosomal
ADLQ: anti-doping laboratories in Qatar
FDR: false discovery rate
FMSI, Laboratorio Antidoping: Federazione Medico Sportiva Italiana
GVS: genome variation server
GWAS: genome-wide association studies
HESI-II: high resolution/accurate mass spectrometer interfaced with a heated electrospray ionization
MADD: MAP kinase activating death domain
MAF: minor allele frequency
MVC: maximal voluntary contraction
MYBPC3, myosin binding protein C: cardiac
NR1H3: nuclear receptor subfamily 1 group H member 3
OR: odds ratio
Spi-1: Spi-1 proto-oncogene
UPLC: ultra-performance liquid chromatography
VO_2*max*_: maximal oxygen uptake.
