# Supplementary material for: Genome-Wide Association Study Reveals a Novel Association Between MYBPC3 Gene Polymorphism, Endurance Athlete Status, Aerobic Capacity and Steroid Metabolism
Source: Front Genet. 2020 Jun 16;11:595. doi: 10.3389/fgene.2020.00595 (PMC7308547; doi:10.3389/fgene.2020.00595)
Supplement: Supplementary file 1 [file Table_1.DOCX]

**Table S1.** List of genes in eQTL with rs1052373 in the blood including their function and associated diseases.

| **SNP** | **Minor Allele** | **Gene name** | **P-value** | **Gene Function** | **Associated diseases** |
| --- | --- | --- | --- | --- | --- |
| rs1052373  TT | T | Spi-1 (Spi-1 Proto-Oncogene) | 3.3251 x10^-69^ | An ETS-domain transcription factor that activates gene expression during myeloid and B-lymphoid cell development | Inflammatory Diarrhea and Primary Mediastinal B-Cell Lymphoma |
|  |  | Myosin Binding Protein C, Cardiac (MYBPC3) | 1.2009 x10^-59^ | A myosin-associated protein found in the cross-bridge-bearing zone (C region) of A bands in striated muscle. Its phosphorylation modulates cardiac contraction | Cardiomyopathy, Familial Hypertrophic, and Left Ventricular Noncompaction |
|  |  | MAP Kinase Activating Death Domain (MADD) |  | A death domain-containing adaptor protein that interacts with the death domain of TNF-alpha receptor 1 to activate mitogen-activated protein kinase (MAPK) and propagate the apoptotic signal. | Diastolic Heart Failure & cardiac hypertrophy |
|  |  | ACP2 (Acid Phosphatase 2, Lysosomal) | 2.1617 x10^-53^ | A histidine acid phosphatase that hydrolyzes orthophosphoric monoesters to alcohol and phosphate. | Bone structure alterations, lysosomal storage defects, and an increased tendency towards seizures |
|  |  | NR1H3 (Nuclear Receptor Subfamily 1 Group H Member 3) | 4.56 x10^-53^ | A nuclear receptor that works as a key regulator of macrophage function, controlling transcriptional programs involved in lipid homeostasis and inflammation. Plays an important role in the regulation of cholesterol homeostasis. Liver X receptors regulate adrenal steroidogenesis | Multiple Sclerosis and Cerebrotendinous Xanthomatosis. Among its related pathways are Lipoprotein metabolism and Nuclear Receptors in Lipid Metabolism and Toxicity |
