# Supplementary material for: Genome-Wide Association Study Reveals a Novel Association Between MYBPC3 Gene Polymorphism, Endurance Athlete Status, Aerobic Capacity and Steroid Metabolism
Source: Front Genet. 2020 Jun 16;11:595. doi: 10.3389/fgene.2020.00595 (PMC7308547; doi:10.3389/fgene.2020.00595)
Supplement: Supplementary file 2 [file Table_2.DOCX]

**Table S2**. Classification of GWAS participants according to sports classes. Distribution of elite athletes in various categories based on sport type-associated peak dynamic (maximal oxygen uptake percentage; VO_2max_) and peak static (maximal voluntary muscle contraction percentage; MVC) components achieved during competition as described previously (3).
